# Laparoscopic plug removal for femoral nerve colic pain after mesh & plug hernioplasty

**DOI:** 10.1186/s12893-015-0046-9

**Published:** 2015-05-17

**Authors:** Yu Ohkura, Shusuke Haruta, Hisashi Shinohara, Seigi Lee, Yudai Fukui, Nao Kobayashi, Kota Momose, Masaki Ueno, Harushi Udagawa

**Affiliations:** Department of Gastroenterological Surgery, Toranomon Hospital, 2-2-2 Toranomon, Minato-ku, Tokyo 105-8470 Japan

**Keywords:** Inguinal hernia repair, Laparoscopic plug removal, Femoral nerve colic pain, Mesh plug method, Calcification

## Abstract

**Background:**

Inguinal hernias account for 75 % of abdominal wall hernias, with a lifetime risk of 27 % in men and 3 % in women. Major complications are recurrence, chronic pain, and surgical site infection, but their frequency is low. Few studies have reported a calcified mesh causing neuropathy by chronic compression of the femoral nerve after mesh & plug inguinal hernia repair. This is the first report of laparoscopic plug removal for femoral colic due to femoral nerve irritation cause by a calcified plug after mesh & plug inguinal hernia repair.

**Case presentation:**

In July 2013, a 53-year-old man presented to our hospital with a chief complaint of colic pain in the left lower limb while walking. The patient had undergone left inguinal hernia repair about 10 years earlier and reported no chronic pain after the operation. Physical examination revealed a colic pain exacerbated by left thigh movement, especially during flexion, but the patient was pain-free at rest and had no sensory loss. Axial computed tomography and magnetic resonance imaging showed that the inward-projecting plug was extremely close to the femoral nerve. Because of the radicular symptoms and the absence of orthopedic and urological disease, we strongly suspected that the neuralgia was associated with the previous hernia operation and advised exploratory laparotomy, which revealed the plug bulging inward into the abdominal cavity. Moreover, the tip of the plug was firmly calcified and compressing the femoral nerve, which lay just beneath the plug, especially during hip flexion. We explanted the plug and his pain resolved after the operation. The patient remains pain free after 20 months of follow up.

**Conclusion:**

In this study, laparoscopic hernioplasty proved useful for plug removal because laparoscopic instruments can easily grasp perilesional tissue, and laparoscopic approach has the benefit of isolating the plug for removal while preserving the onlay patch, and helpful for restoring peritoneal defects. Laparoscopic plug removal effectively resolved colic pain in the left thigh due to compression of the femoral nerve by a calcified plug.

## Background

Inguinal hernias account for 75 % of abdominal wall hernias, with a lifetime risk of 27 % in men and 3 % in women [1]. The surgical techniques used to manage inguinal hernias are primary open herniorrhaphy, open tension-free hernioplasty, and laparoscopic hernioplasty. Lichtenstein et al. [2] introduced a method of tension-free hernia repair using modern mesh prosthetics in 1986, and this method has since become the most often used technique worldwide and has dramatically reduced the incidence of hernia recurrence. Major complications are recurrence, chronic pain, and surgical site infection, but their frequency is low. Chronic pain has become an important complication and significantly impacts a patient’s quality of life. Persistent post-herniorrhaphy pain affecting daily activities is seen in 5–10 % of patients after open mesh inguinal herniorrhaphy [3]. The characteristics of our patient’s complication were different from those of chronic pain; the patient did not experience chronic pain, but rather an acute sharp pain which appeared suddenly 10 years after inguinal hernia repair. We thought that the laparoscopic (retroperitoneal) approach is facile and provides elegant anatomical visualization free of previous scars. We decided to observe the inside of the abdominal cavity by laparoscopy. There is no report in the past about a calcified mesh causing neuropathy by chronic compression of the femoral nerve after mesh & plug inguinal hernia repair. Here we report a case of colic pain in the left thigh due to compression of the femoral nerve by a calcified plug that disappeared after laparoscopic plug removal.

## Case presentation

In July 2013, a 53-year-old man presented to our hospital with a chief complaint of colic pain in the left lower limb while walking. The patient was 175 cm tall, weighed 87.1 kg, and had a BMI of 28.4. The patient’s past medical history was significant for left indirect inguinal hernia; Gilbert/ Rutkow& Robbins classification was type 2 and Nyhus classification was type 2 [4-7]. The patient had undergone left inguinal hernia repair (mesh plug method) at our hospital just 10 years earlier and reported no pain after the operation. In addition, the patient’s postoperative course was uneventful. We used the visual analog scale (VAS) pain scales; 100 mm vertical lines anchored with “no pain” at the bottom and “worst imaginable pain” at the top. In this scale, 0 mm is “no pain” and 100 mm is “worst imaginable pain”. Physical examination revealed a colic pain exacerbated by left thigh movement; VAS 80–100 mm, especially during flexion; however, the patient was pain free at rest; VAS 0–20 mm and had no sensory deficits. The patient also had no signs of inguinal hernia recurrence. The results of routine blood tests were all within normal limits. We considered radicular symptoms, orthopedic disease, and urological disease as a differential diagnosis, but all were refuted by specialists in the respective departments.

**Fig. 1:**
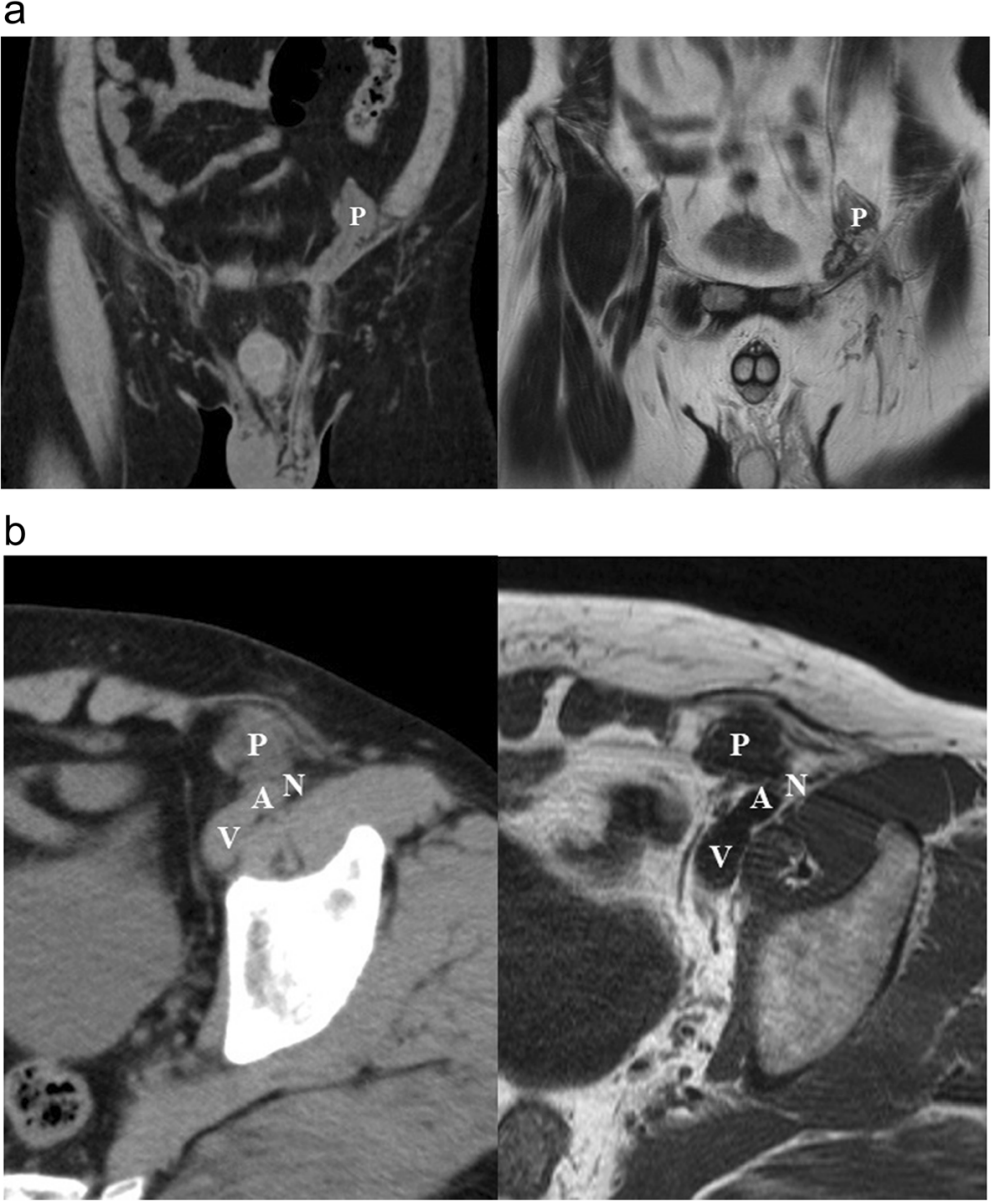
CT and MRI images. **a** Coronal computed tomography (CT) and magnetic resonance imaging (MRI) showing the plug bulging outward into the peritoneal cavity. P: plug. **b** Axial CT and MRI images showing the inward-projecting plug extremely close to the femoral nerve. A: external iliac artery (femoral artery); V: external iliac vein (femoral vein); N: femoral nerve; P: plug

Abdominal ultrasonography was normal. Computed tomography (CT) revealed a low-density structure in the left inguinal region with no evidence of infection (Fig. 1a). This structure showed low signal intensity on T1-weighted magnetic resonance imaging (MRI) and high intensity on T2-weighted MRI (Fig. 1a). CT and MRI also showed the plug bulging outward into the peritoneal cavity, with axial images showing the inward-projecting plug extremely close to the femoral nerve (Fig. 1b).

**Fig. 2:**
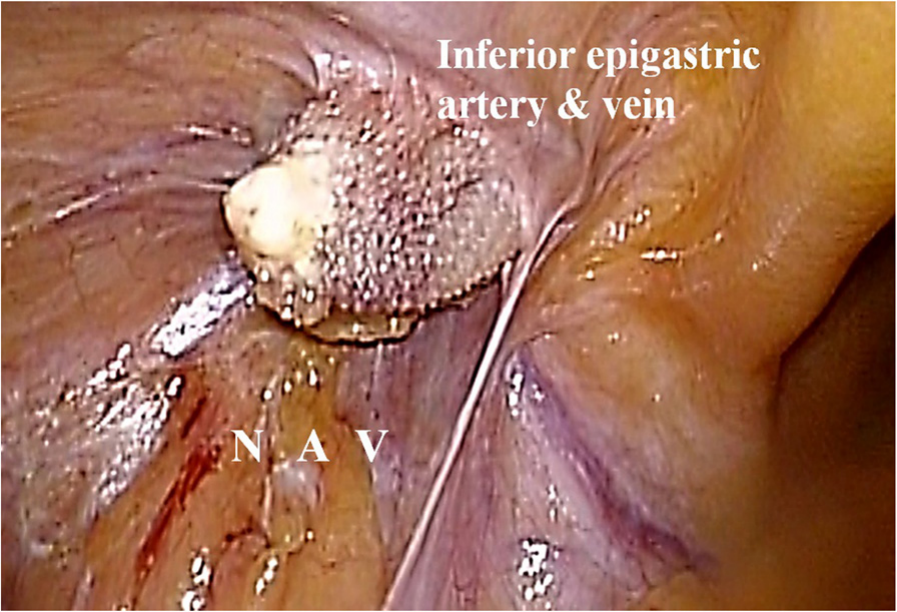
Intraoperative findings. The plug, with a firmly calcified tip, bulging into the abdominal cavity. A: external iliac artery; V: external iliac vein; N: femoral nerve

**Fig. 3:**
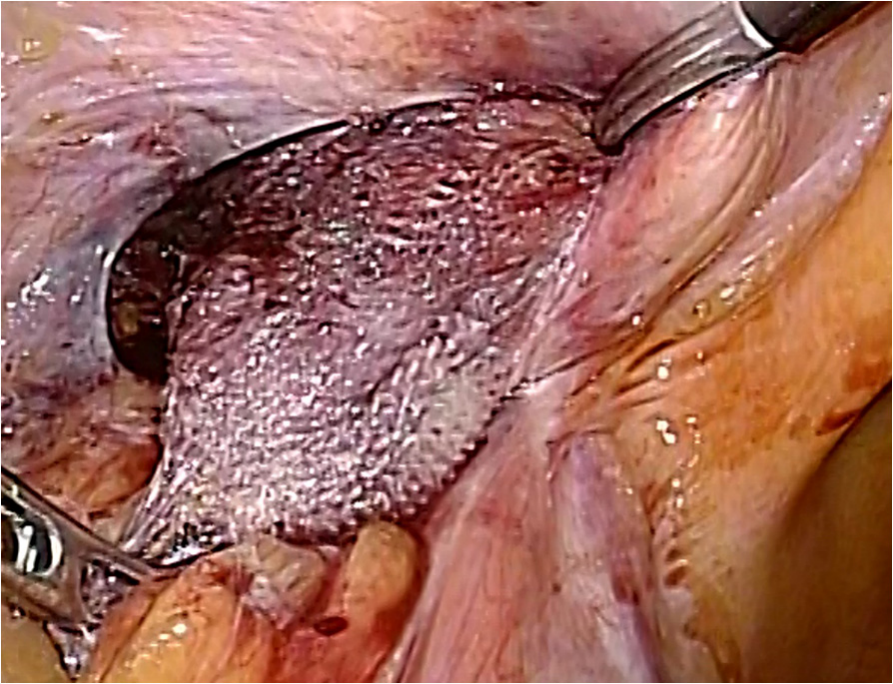
Adhesiolysis. Adhesiolysis was carefully performed between the plug and peritonea, fat, nerve, and vessels

**Fig. 4:**
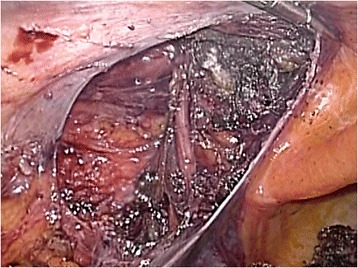
The abdominal wall after plug removal. The peritoneal defect was large and difficult to unite the peritoneum

**Fig. 5:**
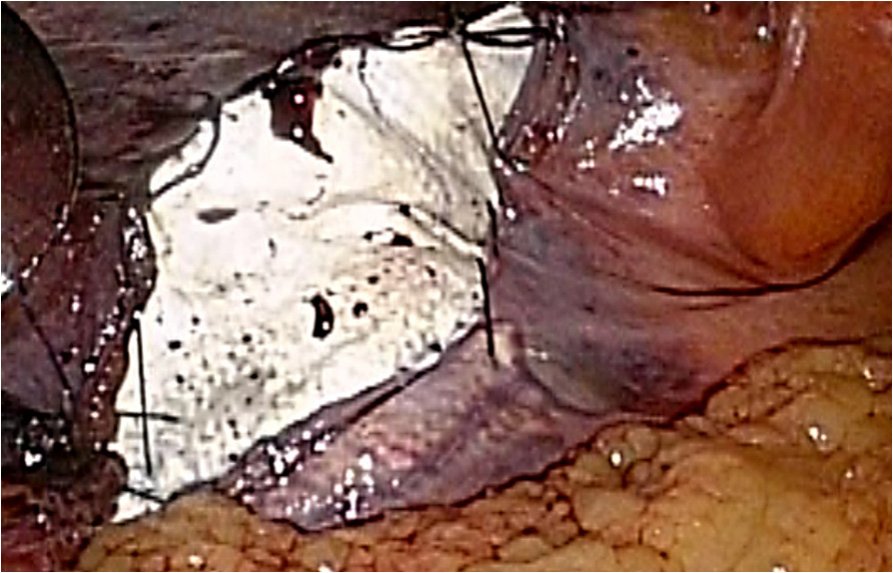
Peritoneal repair. A 12 × 8 cm composite mesh introduced into the pre-peritoneal pocket and positioned over the hernial orifices. Laparoscopic view after plug removal and peritoneal repair

Following neurosurgical, orthopedic, and urologic consultations, we strongly suspected that his neuralgia was associated with the hernia operation 10 years earlier. However, we could not provide a definitive diagnosis preoperatively. Therefore, an exploratory laparoscopy was decided. The first port was inserted through the navel to observe the abdominal cavity. We found that the plug bulged outward into the abdominal cavity and that the tip of the plug had become firmly calcified (Fig. 2). There were no signs of recurrence. The exposed plug was compressing the femoral nerve, which lay just beneath the plug when the patient moved, especially during flexion. We inserted left and right lateral ports and removed the plug laparoscopically. We then carefully broke up adhesions between the plug and peritonea, fat, nerve, and vessels using an ultrasonically activated scalpel (Fig. 3). The abdominal wall after plug removal is shown in Fig. 4. We removed only plug causing leg pain and did not remove onlay mesh. We restored the peritoneal defect after plug removal to prevent future recurrence and adhesions. Once the pre-peritoneal space was prepared, a 12 × 8 cm composite mesh (Ventrio^TM^ Hernia Patch, BIRD Inc.) was positioned to cover the hernia orifice because the peritoneal defect was large and we were not able to unite the peritoneum. The mesh was fixed to the pubic ramus and Cooper’s ligament using endoscopic tackers (Protack®, Covidien Inc.). The superior margins of the mesh were then fixed to the abdominal wall, deliberately avoiding the inferior epigastric vessels. Finally, we sewed the peritoneum to the mesh (Fig. 5).

The patient reported that the sharp pain in his leg disappeared after the procedure; VAS 0–10 mm, and the patient started walking the following day. The postoperative course was uneventful, and the patient was discharged from the hospital 3 days postoperatively. The patient has remained pain-free for 20 months.

## Discussion

Since the first true herniorrhaphy was performed by Bassini in 1887 [8], all modifications and surgical techniques have shared a common disadvantage: suture line tension. Briefly, we discuss the anatomic, physiologic, and pathologic characteristics of hernia recurrence. The prime etiologic factor behind most herniorrhaphy failures is the suturing together, under tension, of structures that are not normally in apposition [9]. Since Lichtenstein et al. [2] introduced a tension-free hernia repair method using modern mesh prosthetics in 1986, it has become the most frequently used technique worldwide and dramatically reduced the incidence of hernia recurrence. They used a cylindrical Marlex mesh plug for the treatment of inguinal and femoral hernias and fixed the mesh with single sutures. Lichtenstein et al. published data on 1,000 operations involving the Marlex mesh plug without any recurrences noted within 5 years of surgery [2]. Furthermore, in a review of 32,206 hernias from the Swedish Hernia Registry and Danish Hernia Database, the overall recurrence rate was only 0.7 % for Lichtenstein repair [10]. Donati et al. [11] reported that only 0.2 % of patients treated by the mesh & plug inguinal hernia repair method developed a recurrence.

Chronic pain after inguinal hernia repair has become an important complication and significantly impacts a patient’s quality of life [12]. Persistent post-herniorrhaphy pain affecting daily activities is seen in 5–10 % of patients after open mesh inguinal herniorrhaphy [3]. Injury to ≥1 of the 3 inguinal nerves (i.e., genitofemoral, iliohypogastric, or ilioinguinal nerve) is considered an important prerequisite for the development of persistent post-herniorrhaphy pain. Therefore, avoiding nerve entrapment and injury is critical. Of all three nerves, it is relatively easy to identify the iliohypogastric nerve which run the front of internal abdominal oblique muscle, and ilioinguinal nerve which run the front of cremaster muscle. Identification of genitofemoral nerve is the most difficult because the genital branch of genitofemoral nerve was very thin. However the genital branch of the genitofemoral nerve is always next to the external spermatic vein, so-called blue line, which is situated along the inferior border of the spermatic cord so that the preservation of the vein means the preservation of the nerve itself. The current consensus is that routine identification and preservation of these nerves is the best means of protection [13]. The first-line choice treatments are anti-inflammatories, nerve blocks, and neuromodulators [14], and pain often resolves with conservative measures. Following complete evaluation of the patient and unsuccessful attempts at non-surgical treatment, surgery may be considered, for examples, mesh removal or the triple neurectomy. Triple neurectomy of the ilioinguinal, iliohypogastric, and genitofemoral nerves is a viable treatment option. In the recent report, the laparoscopic (retroperitoneal) approach provides a facile method to reach the nerves (the ilioinguinal, iliohypogastric and genitofemoral nerves) in 1 stage and also provides a dissection field free of previous scars [15, 16]. Therefore, generally, laparoscopic approach is more recommended than anterior approach. If operative repair is chosen, mesh removal should only be considered as a last resort because firm adhesion is extremely difficult to accomplish. We should remove only plug causing leg pain and should not remove onlay mesh because of the risk of bleeding and nerve injury when we broke up adhesions between the onlay mesh and the surrounding tissue.

The characteristics of our patient’s complication were different from those of chronic pain; he did not experience chronic pain, but rather an acute sharp pain which appeared suddenly 10 years after inguinal hernia repair. Because radicular symptoms, orthopedic disease, and urological disease were refuted as a cause of the sharp pain in the left inferior limb, we strongly suspected that his neuralgia was associated with the hernia operation 10 years earlier [17, 18]. We thought that the laparoscopic (retroperitoneal) approach is facile and provides elegant anatomical visualization free of previous scars. Therefore, we decided to observe the inside of the abdominal cavity by laparoscopy.

Intraoperative finding showed that the plug bulged outward into the abdominal cavity, and moreover, that the tip of the plug had become firmly calcified. In addition, the tip of the plug projected outward. Therefore, we considered that the firmly calcified plug (Meshoma) was suppressing the femoral nerve when the patient moved, especially during flexion. It is unusual that neurological symptoms arise only in response to a plug. In our case, however, the tip of the plug became firmly calcified. Klosterhalfen et al. described calcification of mesh [19] in which calcification and bridging of the mesh pores with fibrous scar tissue was noted. They also reported no complications associated with calcification.

In our case, the cause of calcification was thought to be related to inflammation between the fibrous tissue filling the entire pore and the nylon yarn used to fix the plug tip to the peritoneum. The sharp pain in our patient’s leg disappeared after plug removal. It is extremely rare that the femoral nerve is the cause of post-herniorrhaphy neuralgia, and there is no precedent for our case [10, 20, 21]. Donati et al. [10] examined complications of mesh & plug inguinal hernia repair in 2,902 patients, but none of their reported symptoms were seen in our case. Therefore, the present case is likely the first report of laparoscopic plug removal for femoral colic due to femoral nerve suppression by the plug 10 years after inguinal hernia repair by the mesh plug method.

## Conclusion

In this study, laparoscopic herniorrhaphy proved useful for plug removal and inguinal hernia repair. Laparoscopic instruments can easily grasp perilesional tissue, and the laparoscopic approach has the benefit of isolating the plug for removal while preserving the onlay patch. Moreover, laparoscopic operation is helpful for restoring peritoneal defects. In our case, just to be safe, we restored the peritoneal defect using composite mesh, to reduce the risk of recurrence and adhesion. Laparoscopic plug removal effectively resolved shooting pain in the left thigh due to compression of the femoral nerve by a calcified plug.

## Consent

Written informed consent was obtained from the patient for publication of this case report and any accompanying images. A copy of the written consent is available for review by the Editor of this journal.

## Abbreviations

CT: Computed tomography
MRI: Magnetic resonance imaging
A: External iliac artery (femoral artery)
V: External iliac vein (femoral vein)
N: Femoral nerve
P: Plug

## References

[1] Jenkins JT, O’Dwyer PJ (2008). Inguinal hernias. BMJ.

[2] Lichtenstein IL, Shulman AG (1986). Ambulatory outpatient hernia surgery. Including a new concept, introducing tension-free repair. Int Surg.

[3] Nienhuijs S, Staal E, Strobbe L, Rosman C, Groenewoud H, Bleichrodt R (2007). Chronic pain after mesh repair of inguinal hernia: a systematic review. Am J Surg.

[4] Rutkow IM, Robbins AW (1993). “Tension-free” inguinal herniarrhaphy: a preliminary report on the “mesh plug” technique. Surgery.

[5] Robbins AW, Rutkow IM (1998). Mesh plugs repair and groin hernia surgery. Surg Clin N Am.

[6] Robyn Mitchell G, Gardener RM, Boyd CR (2008). Examining modern approaches to inguinal and femoral herniorrhaphy. JAAPA.

[7] Nyhus LK, Klein SM, Rogers FB (1991). Inguinal hernia. Curr Probl Surg.

[8] Gray SH, Hawn MT, Itani KM (2008). Surgical progress in Inguinal and ventral incisional hernia repair. Surg Clin N Am.

[9] Lichtenstein IL, Shulman AG, Amid PK, Montllor MM (1989). The tension-free hernioplasty. Am J Surg.

[10] Bay-Nielsen M, Nordin P, Nilsson E, Kehlet H (2001). Operative findings in recurrent hernia after a Lichtenstein procedure. Am J Surg.

[11] Donati M, Brancato G, Giglio A, Biondi A, Basile F, Donati A (2013). Incidence of pain after inguinal hernia repair in the elderly. A retrospective historical cohort evaluation of 18-years’ experience with a mesh & plug inguinal hernia repair method on about 3000 patients. BMC Surg.

[12] Fang Z, Zhou J, Ren F, Liu D (2014). Self-gripping mesh versus sutured mesh in open inguinal hernia repair: system review and meta-analysis. Am J Surg.

[13] Alfieri S, Amid PK, Campanelli G, Izard G, Kehlet H, Wijsmuller AR (2011). International guidelines for prevention and management of post-operative chronic pain following inguinal hernia surgery. Hernia.

[14] Ferzli George S, Edwards Eric D, Khoury GE (2007). Chronic pain after inguinal herniorrhaphy. J Am Coll Surg.

[15] Song JW, Wolf JS, McGillicuddy JE, Bhangoo S, Yang LJ (2011). Laparoscopic triple neurectomy for intractable groin pain: technical report of 3 cases. Neurosurgery.

[16] Mahan MA, Kader AK, Brown JM (2014). Robot-assisted triple neurectomy for iatrogenic inguinal pain: a technical note. Acta Neurochir.

[17] Bachul P, Tomaszewski KA, Kratochwil M, Solecki R, Walocha JA (2013). Anatomic variability of groin innervation. Folia Morphol.

[18] Celebi S, Aksoy D, Cevik B, Yildiz A, Kurt S, Dokucu AI (2013). An electrophysiologic evaluation of whether open andlaparoscopic techniques used in pediatric inguinal herniarepairs affect the genitofemoral nerve. J Pediatric Surg.

[19] Klosterhalfen B, Klinge U (2013). Retrieval study at 623 human mesh explants made of polypropylene - impact of mesh class and indication for mesh removal on tissue reaction. J Biomed Mater Res B Appl Biomater.

[20] Meyer A, Blanc P, Balique JG, Kitamura M, Juan RT, Delacoste F (2013). Laparoscopic totally extraperitoneal inguinal hernia repair. Twenty-seven serious complications after 4565 consecutive operations. Rev Col Bras Cir.

[21] Saleh F, Okrainec A, D’Souza N, Kwong J, Jackson TD (2014). Safety of laparoscopic and open approaches for repair of the unilateral primary inguinal hernia: an analysis of short-term outcomes. Am J Surg.

